# Supplementary material for: The Development of an Ultra-Performance Liquid Chromatography–Tandem Mass Spectrometry Method for Biogenic Amines in Fish Samples
Source: Molecules. 2022 Dec 26;28(1):184. doi: 10.3390/molecules28010184 (PMC9822501; doi:10.3390/molecules28010184)
Supplement: Supplementary file 1 [file molecules-28-00184-s001.zip › molecules-2034941-supplementary.pdf]

Supplementary Materials

The Development of an Ultra-Performance Liquid Chromatography–Tandem Mass Spectrometry Method for Biogenic Amines in Fish Samples

Tong Li, Ruiguo Wang and Peilong Wang *

Institute of Quality Standards and Testing Technology for Agro-Products, Chinese Academy of Agricultural Sciences, Beijing 10081, China; litong@caas.cn (T.L.); wangruiguo@caas.cn (R.W.)
* Correspondence: wangpeilong@caas.cn; Tel.: +86-10-82106582

Table S1. Extraction recoveries of 14 BAs derivatives with different extraction reagents. FA means formic acid, AA means acetic acid, ACN means acetonitrile.

Name	extraction reagents				
	ACN	0.1% FA in ACN	0.5% FA in ACN	0.1% AA in ACN	0.5% FA in ACN
Histamine	55.1 ± 7.4%	80.1 ± 4.7%	82.4 ± 5.6%	44.2 ± 2.6%	80.1 ± 4.7%
Tyramine	32.1 ± 4.8%	75.2 ± 5.1%	76.1 ± 7.5%	52.1 ± 7.5%	75.2 ± 5.1%
Dopamine	28.9 ± 8.1%	91.3 ± 10.7%	91.2 ± 7.1%	48.2 ± 8.2%	91.3 ± 10.7%
Tryptamine	38.3 ± 5.2%	78.8 ± 4.2%	79.0 ± 11.8%	68.1 ± 12.4%	78.8 ± 4.2%
Serotonin	64.5 ± 7.4%	89.2 ± 6.1%	82.5 ± 13.0%	33.3 ± 10.0%	86.5 ± 7.7%
Putrescine	22.1 ± 8.2%	93.1 ± 3.4%	92.3 ± 6.7%	26.4 ± 12.0%	82.9 ± 13.4%
Spermine	27.3 ± 11.4%	92.1 ± 6.4%	84.2 ± 2.6%	44.2 ± 2.6%	90.4 ± 8.5%
Spermidine	61.2 ± 9.8%	75.2 ± 8.8%	78.1 ± 8.3%	31.7 ± 17.2%	69.3 ± 12.3%
Octopamine	32.1 ± 10.1%	91.6 ± 0.8%	91.2 ± 9.5%	29.2 ± 10.4%	90.4 ± 5.6%
Benzylamine	58.3 ± 9.2%	88.8 ± 6.2%	88.1 ± 11.0%	60.2 ± 9.2%	78.8 ± 2.6%
1-Phenylethanamine	33.5 ± 7.9%	89.2 ± 7.2%	92.5 ± 12.7%	62.5 ± 3.7%	82.7 ± 4.7%
Cadaverine	17.7 ± 8.6%	90.1 ± 8.4%	84.3 ± 6.3%	14.3 ± 8.2%	92.2 ± 6.4%
2-Phenethylamine	21.8 ± 17.5%	81.3 ± 5.7%	76.4 ± 16.2%	55.5 ± 12.7%	82.7 ± 10.4%
Agmatine	9.3 ± 15.3%	77.2 ± 6.4%	74.3 ± 5.7%	54.3 ± 6.3%	72.5 ± 9.4%

Table S2. Sensitivities of BAs (1000 nM) standard solution derived with benzoyl chloride, dansyl chloride and pyridine-3-sulfonyl chloride.

Name	derivatization reagents		
	benzoyl chloride	dansyl chloride	pyridine-3-sulfonyl chloride
Histamine	4.66×10^6	1.29×10^6	9.27×10^5
Tyramine	1.05×10^7	4.93×10^6	1.36×10^6
Dopamine	1.37×10^7	9.38×10^5	8.03×10^5
Tryptamine	1.62×10^7	7.39×10^6	1.07×10^5
Serotonin	5.15×10^6	3.87×10^6	1.36×10^6
Putrescine	2.70×10^6	1.88×10^6	1.08×10^6
Spermine	1.96×10^6	1.96×10^6	1.33×10^6
Spermidine	1.23×10^6	1.06×10^6	7.11×10^5
Octopamine	2.70×10^6	2.59×10^6	2.16×10^5
Benzylamine	1.86×10^7	2.93×10^6	3.40×10^5
1-Phenylethanamine	1.91×10^7	7.04×10^5	5.87×10^5
Cadaverine	2.30×10^7	9.97×10^6	7.11×10^5
2-Phenethylamine	2.08×10^7	8.56×10^6	2.72×10^6
Agmatine	1.37×10^7	5.16×10^6	3.40×10^5

Table S3. Response of each BAs derivatives under different conditions (concentration of benzoyl chloride and vortex time) of derivatization.

Name	Concentration of of benzoyl chloride					Reaction time			
	1%	2%	3%	4%	10%	5 min	10 min	20 min	30 min
Histamine	8.40×10^6	1.60×10^7	1.52×10^7	1.20×10^7	8.80×10^6	1.28×10^7	1.40×10^7	1.64×10^7	1.60×10^7
Tyramine	1.80×10^7	4.64×10^7	4.56×10^7	3.60×10^7	3.04×10^7	3.60×10^7	4.64×10^7	4.76×10^7	3.96×10^7
Dopamine	2.16×10^7	5.12×10^7	4.84×10^7	4.16×10^7	2.40×10^7	3.00×10^7	3.44×10^7	5.12×10^7	4.04×10^7
Tryptamine	2.68×10^7	5.60×10^7	3.96×10^7	3.56×10^7	3.04×10^7	3.60×10^7	4.88×10^7	5.56×10^7	5.60×10^7
Serotonin	8.40×10^6	1.76×10^7	1.76×10^7	1.64×10^7	1.28×10^7	4.00×10^6	6.40×10^6	1.80×10^7	1.84×10^7
Putrescine	4.80×10^6	1.76×10^7	1.36×10^7	8.00×10^6	4.40×10^6	8.80×10^6	1.56×10^7	1.68×10^7	1.76×10^7
Spermine	2.40×10^6	1.64×10^7	6.40×10^6	3.60×10^6	3.60×10^6	6.80×10^6	1.32×10^7	1.76×10^7	1.68×10^7
Spermidine	2.80×10^6	1.68×10^7	1.56×10^7	3.20×10^6	3.20×10^6	1.12×10^7	1.68×10^7	1.80×10^7	1.52×10^7
Octopamine	3.60×10^6	1.56×10^7	1.28×10^7	2.00×10^6	1.60×10^6	6.40×10^6	1.56×10^7	1.60×10^7	1.52×10^7
Benzylamine	3.08×10^7	6.24×10^7	5.64×10^7	4.80×10^6	2.60×10^7	3.40×10^7	4.92×10^7	4.96×10^7	4.88×10^7
1-Phenylethanamine	3.08×10^7	4.80×10^7	4.32×10^7	3.52×10^6	2.24×10^7	3.64×10^7	4.80×10^7	4.84×10^7	4.68×10^7
Cadaverine	3.92×10^7	6.40×10^7	5.16×10^7	3.08×10^6	2.68×10^7	3.92×10^7	5.44×10^7	6.44×10^7	6.36×10^7
2-Phenethylamine	3.40×10^7	8.00×10^7	7.92×10^7	6.80×10^6	3.60×10^7	3.40×10^7	8.00×10^7	8.44×10^7	8.12×10^7
Agmatine	2.24×10^7	3.20×10^7	2.64×10^7	1.60×10^6	8.40×10^6	2.24×10^7	3.20×10^7	3.24×10^7	3.04×10^7

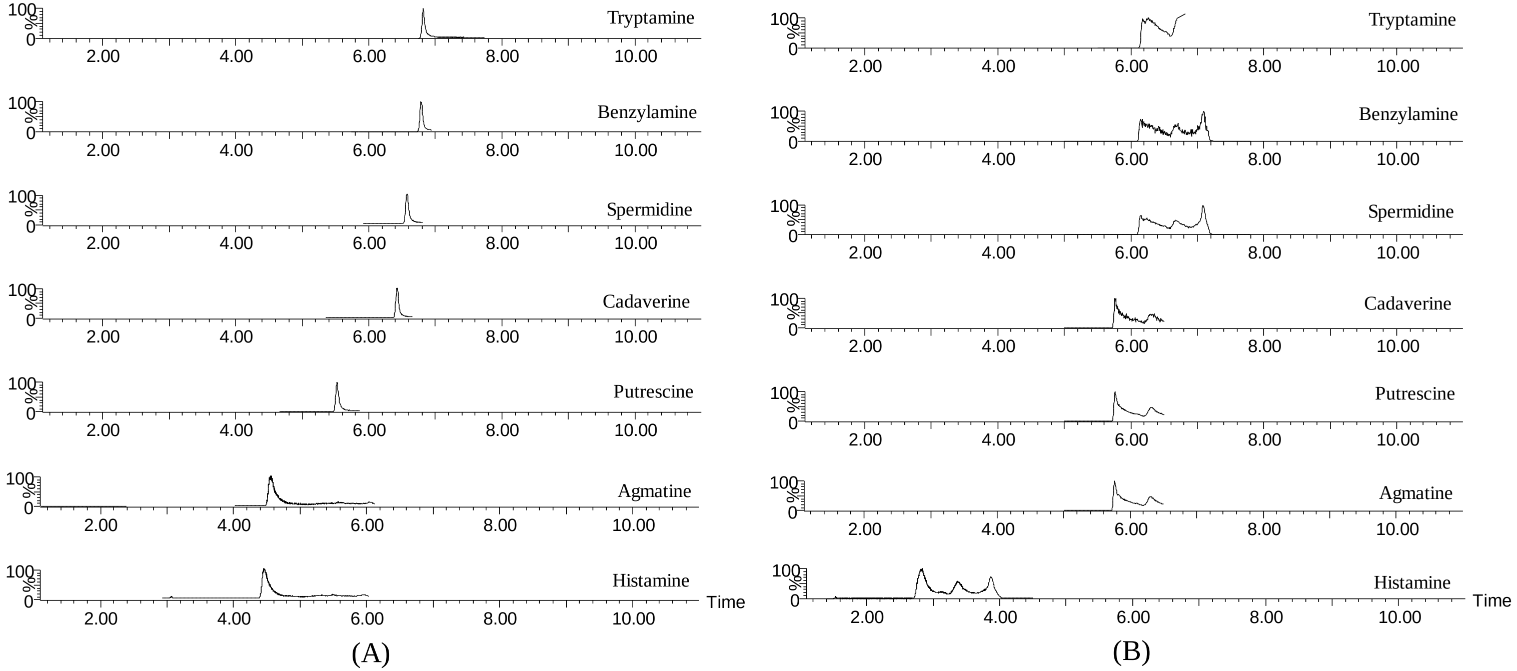

Figure S1. Chromatogram of 7 BAs derivatives in 1000 nM standards mixture. Results show that the chromatographic peak is obviously broadened or even trailing, when the flow rate was 0.2 mL/min (Figure S1A). Chromatography peak shapes were unsatisfactory, when the mobile phase A was water and phase B was acetonitrile (Figure S1B).

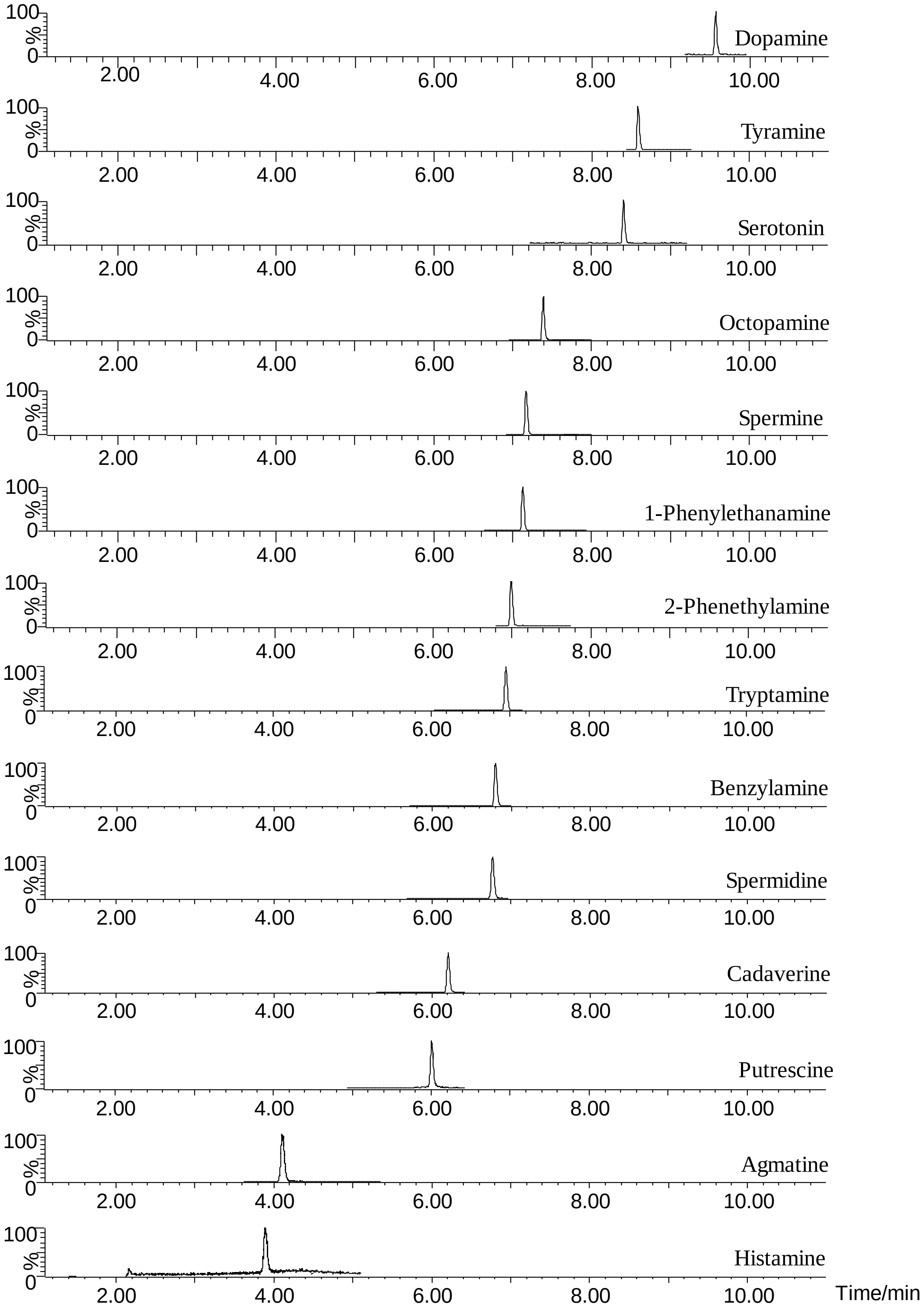

Figure S2. Chromatogram of 14 derivatized BAs in spiked fish sample (1000 nM).

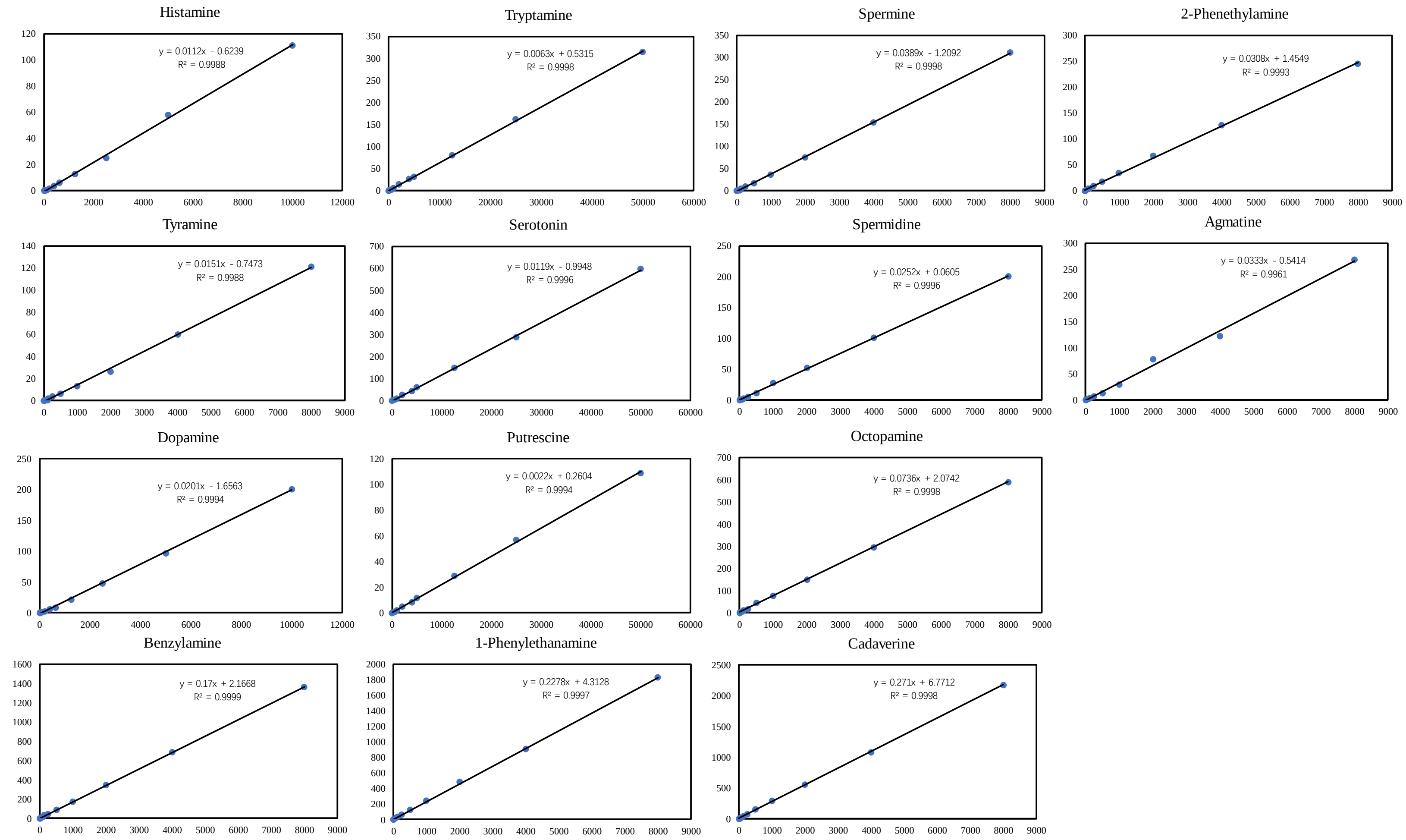

Figure S3. Graphs of calibration curves with data points of 14 BAs derivatives.

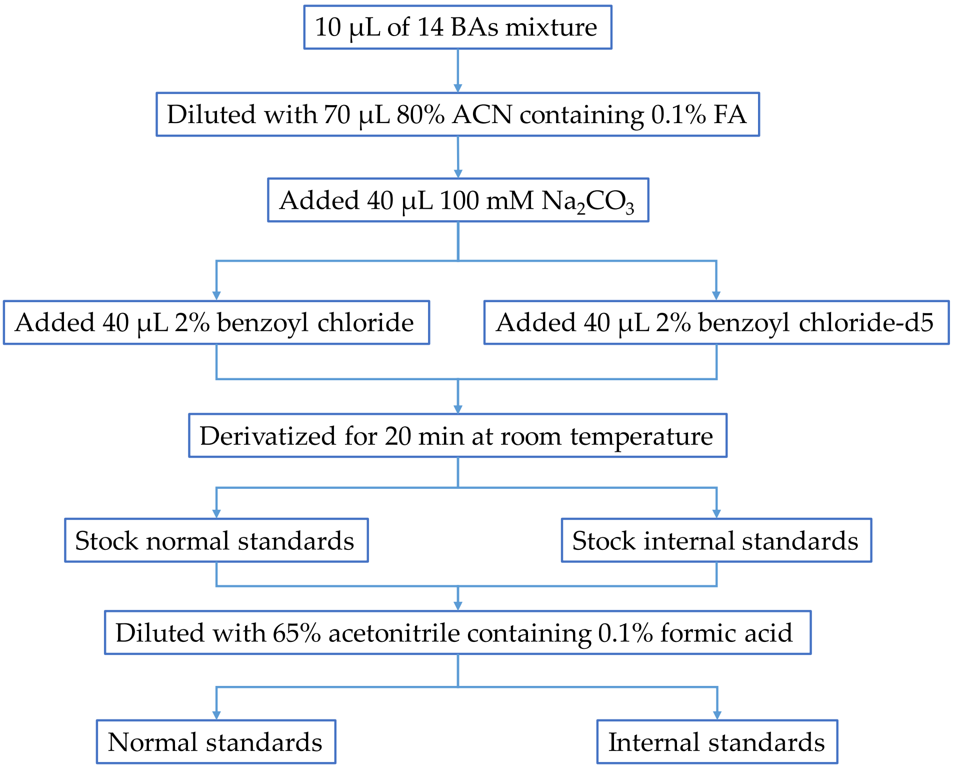

Figure S4. A flow chart for the steps of derivatization of standards.

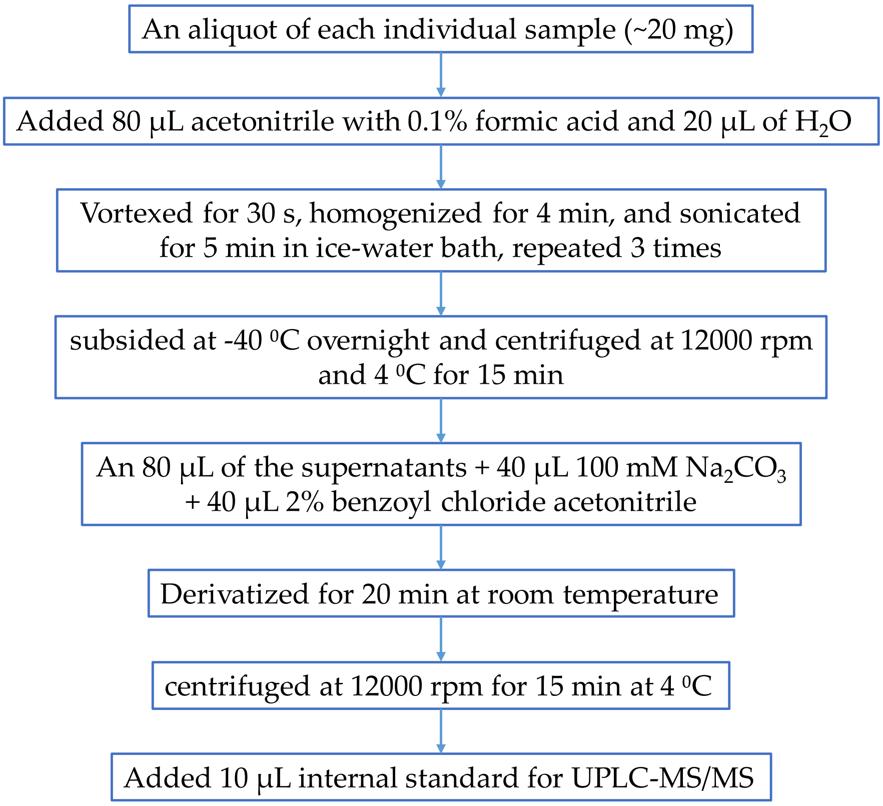

Figure S5. A flow chart for the steps of derivatization of fish samples.
